# Stakeholders' Views on E-cigarette Legislation: A Qualitative Study in Taiwan

**DOI:** 10.3389/fpubh.2019.00354

**Published:** 2019-11-29

**Authors:** Chin-Shui Shih, Jean-Francois Etter

**Affiliations:** Institute of Global Health, Faculty of Medicine, University of Geneva, Geneva, Switzerland

**Keywords:** e-cigarette, stakeholders, qualitative, smoking, legislation

## Abstract

**Objectives:** Little is known about stakeholders' opinions on e-cigarette legislation in Taiwan. Our aim is to understand the perspectives of stakeholders regarding the current legal system and measures that could be included in future e-cigarette legislation in Taiwan.

**Materials and Methods:** We conducted in-depth interviews with 14 Taiwanese stakeholders, using semi-structured questionnaires, either face-to-face or via telephone, in 2016–2017. All interviews were transcribed.

**Results:** The current legal system is applied to e-cigarettes even though it does not mention them specifically, this system carries risks and faces challenges from anti-tobacco and vaper groups. Some weaknesses in the current legislative framework were noted, including the facts that e-cigarettes are sold without government approval, that there are no manufacturing standards, no inspections or monitoring, and no regulations for usage or advertising. There was wide acceptance among stakeholders that e-cigarettes should be better regulated, particularly e-cigarettes containing nicotine. Most interviewees agreed that there is a need to restrict e-cigarette use by teenagers and in public places where tobacco smoking is prohibited. Most interviewees also would like to restrict marketing, ban sales in vending machines, via mail order, and over the internet; label content and nicotine levels; and introduce health warnings and taxation.

**Conclusions:** Almost all interviewees agreed that there is a need for a specific legal framework for e-cigarettes in Taiwan, and that e-cigarettes should be regulated in the same way as combustible cigarettes.

## Introduction

It is unclear how to best regulate e-cigarettes. In 2019, 98 countries had national or federal laws regulating e-cigarettes, including laws related to their sale, advertisement, packaging, product regulation, reporting, taxation, use, and classification (1). Banning e-cigarette use in public places has received public support in some European countries (2) and in the USA (3).

Taiwan has had a legal framework for tobacco smoking for over 20 years. Taiwan's Tobacco Hazards Prevention Act (THPA) was adopted and implemented in 1997, and subsequently amended in 2000, 2007, and 2009 (4). In 2009, the authorities decided to use these existing laws to regulate e-cigarettes. E-cigarettes need to meet requirements under the Pharmaceutical Affairs Act (PAA) to be approved by the Taiwanese Food and Drug Administration (TFDA) for manufacturing, importing, and selling. No e-cigarette application has been filed and none received this approval. Selling a nicotine-free e-cigarette violates Article 14 of the THPA, and claim that a nicotine-free e-cigarette has medicinal effects will subject their authors to a fine under the PAA. However, recent court decisions (5–8) have challenged the appropriateness of applying Article 14 of the THPA to e-cigarettes.

E-cigarette use is still not common among the general population in Taiwan (prevalence 2.7%), but its prevalence is higher among current smokers (14.2%) and youths (7%) (9), even though these products are illegal. E-cigarette users in Taiwan purchase e-cigarette via the Internet or in stores (10) that claim selling nicotine-free liquids. At the time of the end of our study, there was no existing law explicitly applying to e-cigarettes in Taiwan: the term “electronic cigarettes” or “e-cigarettes” did not appear in any acts or laws. The health authorities relied on explanatory notes derived from existing laws to regulate e-cigarette. Applying existing laws to a new product such as e-cigarette may not be optimal, and this is a matter of concern for health authorities, legislators, anti-tobacco groups, and pro-e-cigarette groups. Consequently, Taiwan is in the process of formulating regulations for e-cigarettes, in particular, by modifying its Tobacco Hazards Prevention Act (11). Taiwanese authorities are faced with the choice of maintaining the current legal situation or adopting specific legislation, as Europe (12), the USA (13), and other countries have done in recent years.

Health authorities and other stakeholders demand further research on e-cigarettes to create a regulatory framework based upon scientific evidence and acceptable and effective measures. Only a few reports have been published on e-cigarette regulations in Taiwan, and most were opinion papers that were not based on original, empirical data (14). In this context, the primary aim of the current qualitative study was to examine the perspectives of various stakeholders, including e-cigarette users, on current e-cigarette legislation in Taiwan, and to collect their suggestions for future policies.

## Materials and Methods

From October 2016 to January 2017, we conducted in-depth, semi-structured interviews with e-cigarette stakeholders residing in Taiwan. Prior to data collection, this study received a “Certificate of REC Approval” from the Research Ethics Committee (REC) of the National Taiwan Normal University in Taiwan.

### Participants

Participants included e-cigarette sellers and users, healthcare workers, academics, anti-tobacco association, vaper organization, and health, education, and pharmaceutical authorities. We used a convenience, as opposed to a representative sample. Participants were main stakeholders affected by e-cigarette issues and were identified by the researchers through personal links or the recommendation of other stakeholders. We recruited e-cigarette users from the websites of Taiwanese vapers associations.

We contacted 20 stakeholders, of whom we interviewed 16; the other four could not be interviewed because of their time constraints. Two of the 16 we interviewed later asked to be excluded for personal reasons. Among the 14 participants included in the current analysis, there were six females and eight males. Six were e-cigarette users. Five signed an informed consent form, while nine people, including five interviewed by telephone agreed verbally to be interviewed. Their characteristics are summarized in Table 1.

**Table 1:** Characteristics of stakeholders interviewed in Taiwan.

**Category**	**Gender**		**University or Post-graduate**	**Interview duration average in minutes**	**Sub-total**
	**Male**	**Female**			
Authority (Health and Education)	0	4	4	58	4
Expert (Law and Health)	1	1	2	50	2
Physician	1	0	1	74	1
Anti-tobacco association	0	1	1	53	1
Vape association^+^	1	0	N.A.	31	1
Merchant^+^	3	0	N.A.	60	3
Vaper^+^	2	0	2	42	2
Total	8	6	10	54	14

+ *E-cigarette users*.

### Independence and Neutrality of the Project

The participants who agreed to be interviewed were informed that we would not disclose their identity, that this study was a scientific study without financial or other support of any agency in Taiwan or Switzerland, and that it involved no industry; retailers; manufacturers of medical equipment, electronic cigarettes, or e-liquid; tobacco companies; or governmental authorities. The authors do not have any conflict of interest.

### Stakeholder Interviews

All interviews were conducted in Chinese by the first author, primarily face-to-face (*n* = 9) or by telephone/mobile phone (*n* = 5). Face-to-face interviews were conducted at a location selected by stakeholders. Eight participants in face-to-face interviews agreed to be recorded, except the merchant/vaper. All recorded interviews were later transcribed. The five phone interviews were not tape-recorded because of a technical problem, but manual transcriptions, some verbatim, were taken during the interview.

The semi-structured questionnaires used for the interviews consisted of standard questions, asked of all stakeholders, and stakeholder-specific questions. Questions addressed both existing measures in THPA and potential legislation options for e-cigarettes. Before we conducted the interviews, we pre-tested the questionnaire among volunteers (*n* = 5), including e-cigarette users and non-users. The standard questions, covered three general areas. Part 1 included two questions on current regulations namely PAA and THPA; Part 2 included 10 questions on various control measures currently enforced by the THPA or in other countries, that included expanding e-cigarette access channels (selling in vending machines, via mail order, and over the Internet), taxation, labels informing on content and nicotine level, maximum nicotine allowable level, warning labels, restrictions on advertising, age limitation, and bans on use in public places; Part 3 included four questions comparing electronic and combustible cigarettes. The stakeholder-specific questions on future legislation were tailored to the interviewees, who included authorities, physicians, anti-smoking non-governmental organizations (NGOs), merchants, e-cigarette users, and academics.

### Data Analysis

Qualitative content analysis was performed (15, 16). Each question in our semi-structured questionnaires targeted a single topic. After we transcribed the interviews, we read through the transcripts, and coded the transcriptions manually for each topic. The codes were sorted according to their similarity and then organized together to generate themes.

We integrated the first two standard questions (Part 1) and the stakeholder-specific questions together during analysis. Following these steps, we derived categories and assigned them appropriate labels. Each question in Part two of the standard questions addressed a single topic and was analyzed as such. Participants indicated whether or not they supported each of a number of legislation measures and then were asked to justify their decision. Categories were generated within each justification topic. We also provided quantitative data on support for each tobacco-control measure.

We categorized and organized our findings into four main sections. Selected quotations are presented here as either evidence or for explanatory purposes.

## Results

### The Current Legal System Carries Risks and Facing Challenges From Anti-tobacco and Vaper Groups

Four main issues concerning the current legal system were highlighted. First, stakeholders cited problems pertaining to compliance with the law. According to the Ministry of Health and Welfare, e-cigarettes need approval by the TFDA.

“*E-cigarette liquid containing nicotine is regulated by PAA but the device or atomizer is not regulated. However, to date, no application was filed to the TFDA so no e-cigarette has been approved by* TFDA.”

That is to say, even though e-cigarettes cannot be legally sold or used in Taiwan, they are routinely found at the border, sold in shops, and used.

Second, stakeholders identified weaknesses that need to be fixed. “*The THPA was enacted in an era before e-cigarettes*,” according to the HPA (Health Promotion Administration), so there is no mention of e-cigarettes in the THPA. The PAA has standards for manufacturing, importing, inspecting, and monitoring to assure the safety, effectiveness and quality of pharmaceuticals; but, as one vaper who is also a store owner stated:

“*No specific standards have been written for e-cigarette manufacturers and merchants to follow.” “Also, the use of Article 14 of the THPA has been challenged by a company in district court; and the court ruled in favor of the company and proposed that the Ministry of Health and Welfare should amend the THPA to include e-cigarettes.”*

Third, stakeholders said that the current legal system fails to properly regulate e-cigarette use and sales.

“*There is no regulations for e-cigarette use,”* said one health official, driving most e-cigarette use underground.

“*You can't do anything to anyone who carries or uses e-cigarettes at university campus. Under current legislation, the policeman if informed would trace the e-cigarette source rather fine or punish the user,”* said one expert.

Fourth, stakeholders pointed out that there seems to be faulty logic in the regulations. As one legal expert stated:

“*E-cigarette regulation by the PAA is not coherent, because e-cigarettes are not medication for treating diseases or curing illness. E-cigarettes are used for leisure or addiction, which means they are not pharmaceuticals to treat diseases or cure illness, as proposed by the FAA*.”

### E-cigarettes Are Different Than Conventional Tobacco Cigarettes

As one e-cigarette user said, e-cigarette differ from tobacco cigarette.

“*E-cigarettes are not tobacco; e-cigarettes are different from tobacco cigarettes; e-cigarettes produce vapors that don't contain toxic substances found in tobacco cigarette smoke*.”

A health authority representative mentioned that this is the reason they cannot apply the THPA to e-cigarettes without amending it.

The differences between electronic and combustible cigarettes mentioned by stakeholders included: (1) E-cigarettes contain no tobacco. (2) E-cigarettes are heated electronically, so vapor is generated without burning or smoke. (3) E-cigarettes, as opposed to combustible cigarettes, are an emerging technology. (4) The contents and nicotine levels in e-cigarettes can be readily adjusted, which is not nearly as easy with tobacco. In addition, e-cigarette merchants noted that there are no additional sales taxes on other products that contain nicotine, like nicotine patches.

### Arguments About Health and Other Effects of E-cigarette

Stakeholders held very different perspectives toward the health effects of e-cigarette; (1) vapers claimed that e-cigarettes generate no secondhand smoke; but other stakeholders expressed concern about potential chemicals in the exhaled vapors. (2) Vapers expressed the opinion that e-cigarettes are not harmful and even have some benefits. However, other stakeholders believed e-cigarettes can adversely affect health. (3) Vapers thought e-cigarettes serve to replace tobacco cigarettes and other sources of nicotine; however, the other interviewees expressed doubts that e-cigarettes help smokers quit tobacco.

The e-cigarette was not recommended to use for tobacco cessation by health authorities so the health professionals won't proactively suggest its use to their patients who smoke.

“*E-cigarette is not endorsed as a cessation tool by health authorities, so it is unethical to suggest smokers its use for reducing or stopping tobacco cigarette. I (a physician) receive many smokers asking about the use of e-cigarette to help stop smoking. In such a case, I would advise with balanced guidance and alerts.”*

### General Comments on Future Legislation

Almost all the interviewees expressed the need for new regulations for nicotine-containing e-cigarettes; and agreed that either a new act is needed or e-cigarette-specific articles should be added to some existing act, like the THPA.

“*The HPA proposed modifying the THPA* *(**17**)* *to include e-cigarettes, as suggested by representatives from multi-sector ministries and civil societies.”*

The revised draft was made available for review from 4 January to 6 March 2017.

#### The Purpose of Legislation Differs Between Merchants and Other Stakeholders

According to e-cigarette merchants, e-cigarette legislation is needed for four reasons: first, so that the product can be sold legally; second, to set norms and product standards for industry, merchants, and vapers to follow; third, to boost the economy and increase tax revenue; and fourth, for safety reasons, to avoid the manufacture or importing of sub-standard products. However, some stakeholders did not totally agree with these statements, and were more in favor of developing a comprehensive legal framework, from product to end user.

#### The Effect of Legislation

E-cigarette merchants raised questions about how new regulations will impact the market. First, they feared that excessively high product standards would be set, and that will benefit international tobacco companies over small manufacturers. Second, they anticipated that product accessibility would increase; for instance, 24-h convenience stores would now sell e-cigarettes. Third, they wondered if some currently-available flavors would become forbidden in e-liquids.

The domestic anti-tobacco group wanted to restrict e-cigarettes for health reasons. They would like to see e-cigarettes, both with and without nicotine, regulated and effective control put in place to prevent teenagers and ex-smokers from using them. They support a dual regulation.

“*Requiring TFDA approval for nicotine-containing e-cigarettes and regulating nicotine-free e-cigarettes under the THPA.”*

The other stakeholders expressed the urgent need to have clear laws based on scientific evidence.

“*We have no way to kick people using e-cigarettes off university campuses, and we need legislative empowerment,”* said one expert.

#### Arguments About Nicotine and Other Health Effects

Nicotine-containing and nicotine-free e-cigarettes were mentioned during the interviews. The interviewees involved in e-cigarette businesses explained that they sell nicotine-free e-liquid legally because its contents are flavored and entail substances that are used widely in the food industry. A legal expert commented:

“*If nicotine is the main concern, there is no regulation needed for nicotine-free e-liquid.”*

The authorities and anti-tobacco groups delivered strong messages about e-liquids. An official said most of e-cigarette contained nicotine though claiming to be nicotine free.

“*More than 70% of e-liquids that tested positive for nicotine came from various sources, including those sold at markets and those confiscated at the border.”*

The other major remarks from stakeholders were that e-cigarette vapors contain carcinogens; e-cigarettes are dangerous in terms of battery explosions and second-hand vapors; and e-liquids are sometimes used to inhale narcotics. They supported regulating nicotine-containing e-cigarettes through the PAA, and nicotine-free ones under the THPA.

#### Creating a Specific Act or Chapter vs. Amending an Existing act

Few stakeholders supported a new law specific for e-cigarettes, as such a legislative process would be time consuming. The reasons for a new law given by experts and included:

“*E-cigarettes are not pharmaceuticals or medicines for curing diseases;” “There is no pharmaceutical ingredients except nicotine for e-cigarettes;” “The standards for pharmaceuticals are far too high for e-cigarettes;” “Their promotional activities are more similar to tobacco cigarettes.”*

Conversely, members of the industry and users preferred a speedy approach, like modifying the THPA.

“*E-cigarettes could be sold, imported and used legally under regulations as soon as possible.”*

One health care representative and authorities said:

“*We are watching other countries, and international actions are an important reference;” “Consensus reached to include e-cigarette by amending Article 2 of THPA;” “An explicit definition of e-cigarette in THPA empowers custom officers.”*

### Opinions on Specific Control Measures

We asked all interviewees to express their opinions on the following specific measures, which are used pursuant to the THPA and are implemented in some other countries where e-cigarettes are used legally (Table 2).

**Table 2:** Stakeholders' response to 10 e-cigarette control measures.

	**Authority**				**Expert**		**Physician**	**Anti-tobacco association**	**Vapers**						**Summary**
									**Association**	**Merchant**			**Users**		
3.4.1	–	–	–	–	–	–	–	–	+/–	+	–	–	+/–	+	10 (–)
3.4.2	–	–	–	–	+	–	–	–	–	–	–	–	+	+	11 (–)
3.4.3	–	–	–	–	–	–	–	–	–	–	–	–	+	+	12 (–)
3.4.4	+	+	+	+	+	+	+	+	–	+	+	+	0	+	12 (+)
3.4.5	+	+	+	+	+	+	+	+	+	+	+	+	+	+	14 (+)
3.4.6	+	+	+	+	+	+	+	+	+	+	+	+	+	+/–	13 (+)
3.4.7	+	+	+	+	+	+	+	+	+	+	–	+	–	+	12 (+)
3.4.8	+	+	+	+	+	+	+	+	+	+	–	0	+	+/–	11 (+)
3.4.9	+	+	+	+	+	+	+	+	+	+	+	+	+	+	14 (+)
3.4.10	+	+	+	+	+	+	+	+	+	+	+	–	+	+	13 (+)

*NB: + “in favor;” - “against;” +/- “neither in favor or against”*.

3.4.1 Selling in vending machine

3.4.2 Selling via mail order

3.4.3 Selling over the internet

3.4.4 E-cigarette taxation

3.4.5 Labels notifying potential buyers of content and nicotine level

3.4.6 Maximum nicotine level and product standards

3.4.7 Warning labels

3.4.8 Restrict advertising

3.4.9 Age limitations

*3.4.10 Ban use in public places where tobacco smoking is prohibited*.

#### Selling in Vending Machines

Banning e-cigarette sales in vending machines was widely supported by stakeholders (10 of 14). Many stakeholders supported applying the same restrictions to vending machines as those set forth in the THPA. Three main concerns were identified: about users, sellers, and society. Regarding users, one e-cigarette promoter stated:

“*Skill and safety are reasons to ban vending machine sales and limit e-cigarette sales to certified stores where users can learn the right way to use them*.”

Second, merchants noted that it is difficult to verify a buyer's age; sales should be restricted to only licensed stores; and access to e-cigarettes should be limited. Third, regarding social factors, the environment was felt to not be conducive to e-cigarette use, in terms of legal compliance and the characteristics of Taiwanese people; a representative from e-cigarette merchants commented:

“*Maybe in 20 years, when the environment matures.”*

Few interviewees supported selling e-cigarettes in vending machines even if, as one supporter said:

“*Sales are limited to nicotine-free e-cigarettes and the buyer's identity or age is checked.”*

#### Selling via Mail Order

Most respondents (11 of 14) did not support selling e-cigarettes by mail order. They cited concerns over products, sellers, and effects. First, regarding products, one expert said:

“*E-cigarettes are worse than conventional tobacco cigarettes, so mail orders should be banned.”*

Second, regarding sellers, many stakeholders agreed that:

“*It is difficult to check buyer's ID and age,” “only licensed stores should sell by mail order,”* and “*more restrictions are needed to limit accessibility.”*

Last, regarding effects, interviewees worried that such easy access would result in increased use.

#### Selling Over the Internet

Most interviewees (12 of 14) did not support selling e-cigarettes via the Internet. The main reasons pertained to the product, sales, and accessibility. First, regarding the product, vapers and sellers raised two points:

“N*o quality assurance,”* and “*risk of short circuits or explosions.”*

Second, regarding sales, besides most stakeholders worried about the difficulty of verifying age, again vapers preferred for e-cigarettes to only be sold at licensed stores, not over the Internet, so new users can learn how to use them properly in stores. Third, regarding accessibility, they wanted to avoid “*giving teenagers easy access*.”

However, one supporter (vaper) argued that the Internet would be not easy to control. Another supporter (e-cigarette merchant) suggested “*limiting sales only to authorized internet sellers.”*

#### E-cigarette Taxation

A specific tax is levied against tobacco products, particularly for health reasons. The government invests the revenue from this tax in healthcare. Almost all interviewees agree that an additional tax should be levied on e-cigarettes, similar to tobacco cigarettes to “*increase government revenue”* said one vaper. The cigarette tax is adjusted from time to time and is currently 20 TD (about 0.65 USD) per pack. Stakeholders suggested applying a steep tax on e-cigarettes to decrease motivation to purchase them.

“*To use the tax revenue for healthcare,” “to set a tax rate for both e-liquids and devices,” and “to match the rate suggested by the WHO; for example, 70% of the e-cigarette's overall price.”*

One person (vaper) held a different opinion, saying:

“*E-cigarettes are a nicotine-replacement product, and the government doesn't tax other similar products, like the nicotine patch.”*

#### Labels Notifying Potential Buyers of Content and Nicotine Levels

All stakeholders agreed that the nicotine level must be indicated on e-liquid packaging. Their reasons included right to know and quality control. One authority representative said:

“*Users have the right to know the contents, just as for other products,” “because other unneeded or even dangerous materials are easily added to the liquid”*

Regarding control measures, interviewees mentioned setting standards, testing, and certifications; using understandable language on labels; and monitoring.

#### Maximum Nicotine Level and Product Standards

All interviewees but one agreed that a maximum authorized nicotine level should be established, but no one cited any scientific basis for a specific maximum level. One vaper said:

“*I don't think setting a maximum level would be useful or meaningful.”*

Labels should allow:

“*Vapers to be aware of nicotine levels as an alert to reduce usage and as a reference to indicate dependence; also to avoid overdose*.”

Another concern expressed related to criteria and standards. An association representative suggested:

“*Regulating by setting criteria and standardized test methods, so manufacturers can follow them to increase product quality.”*

#### Warning Labels

Most stakeholders agreed that there should be warning labels on e-cigarettes, because they are “*addictive.” Moreover*, they argued that

“*The packages will be thrown away, so there should be warning labels on the device or e-liquid container itself.”*

One dealer argued that proper use is more important than warning labels, because e-cigarettes are equipped with electronics. One vaper said that a warning label is necessary for nicotine-containing e-cigarettes, but not for nicotine-free ones, because the latter product is harmless.

#### Advertising

The majority of stakeholders (11 of 14) agreed that the same restrictions should be applied to e-cigarettes that are applied to tobacco products.

“*Normally, commercial advertisements must meet regulations set by the state, not to mention that promoting a sub-standard product would violate regulations.”*

However, stakeholders personally involved in the e-cigarette business had divided views, from “*no advertising, as for tobacco products,”* to “*no advertising for nicotine-containing products, but allowing them for nicotine-free ones,”* to “*a need for advertising for a variety of products.”*

#### Age Limitations

All agreed on prohibiting people under 18 years old from purchasing or using e-cigarettes. The reasons given included: avoiding early addiction; for safety reasons; and avoiding becoming a smoker. The potential impact of this restriction on smokers under 18 was not mentioned.

“*Age limitation is a must, and a penalty is necessary,”* said one expert.

#### Public Places

Almost all (13 of 14) agreed on banning e-cigarette use in public places where tobacco smoking is already prohibited. Their reasons included:

“*So non-smokers (non-vapers) are not bothered”; “to respect non-users”; “big vapors cause a nuisance”;* and “*to apply the same rules for smoke and vapors, since they are almost the same.”*

## Discussion

Almost all our interviewed stakeholders shared the view that a comprehensive, specific legal framework is needed for e-cigarettes in Taiwan. An earlier population-based telephone survey, conducted by the HPA in 2016 (18), yielded similar results, with 91% of respondents agreeing that “*e-cigarette management should be strengthened.”* The opinion expressed by authorities, as well as by anti-tobacco and pro-e-cigarette groups, is that either a new law or amending existing legislation are needed to regulate nicotine-containing e-cigarettes. Nicotine-free e-cigarettes may be regulated differently.

The pro-e-cigarette group argued that e-cigarettes are different from combustible cigarettes and are an alternative rather than similar to cigarettes (19). The Public Health England estimated that e-cigarettes are 95% less harmful than cigarettes and could be a smoking cessation aid (20). However, Taiwanese health authorities do not share this view, believing that e-cigarettes are more harmful to health than estimated by Public Health England.

Drawbacks of current Taiwanese regulations for e-cigarettes were identified. First, the technical standard set for e-cigarettes is as high as for pharmaceuticals, which is viewed as excessive. Theoretically, e-cigarette sales, importation, and distribution are illegal without TFDA approval; but the reality is that e-cigarettes are imported and used by many Taiwanese adults and adolescents. Our interviewees suggested to review the current technical standards and the policies regarding e-cigarette, using scientific evidence and acceptable interventions. Second, a gray area exists, since e-cigarette use is beyond the jurisdiction of the PAA, which regulates product manufacturing, importation, and sales. Taiwanese universities cooperate with the police and the HPA but cannot fine e-cigarette users. Also, self-importation of over-the-counter medicines is allowed under the PAA and e-cigarettes are not on the prohibited list (21). In the end, customs officers have no legal right to confiscate self-imported e-cigarettes. Lastly, a district court has ruled that regulating nicotine-free e-cigarettes under Article 14 of the THPA is questionable. A person who imported coils was fined based on the Article 14 of THPA on the reason that the imported coils were components of e-cigarettes. The case was overruled by the determination of district court (7) as the coils themselves would not lead to e-cigarette use. Adequate and explicit regulations addressing the health effects and safety of e-cigarettes are needed that are appropriate for Taiwanese culture and behaviors, and that have the ability to reduce smoking-related mortality and morbidity.

We anticipate that Taiwan will move soon to strengthen its e-cigarette policies through legal interventions. State interventions based on explanatory notes derived from pre-existing laws will be replaced by interventions based on new, specific laws, and the issue of the lack of rules on the use, implementation and compliance of e-cigarettes will be fixed by the future legal system. Consequently, the various authorities will be legally empowered to implement the relevant e-cigarette articles under the amended THPA.

In 2016, the Conference of the Parties (COP)7/Framework Convention on Tobacco Control (FCTC) welcomed a WHO report inviting Parties to consider regulatory measures for e-cigarette to prevent the initiation by non-smokers and youth, minimize potential health risks for users and protect non-users from exposure to their emissions, prevent unproven health claims, and protect tobacco control activities from all commercial and other vested interests (22, 23). However, the WHO, the COP/FCTC, and the FCTC Secretariat take a very negative stance against e-cigarettes and reduced-risk products, and may not offer the best guidance for legislation. Stifling competing products with excessive restrictions may have the unintended consequence of shielding the combustible cigarette market from competition (23, 24).

The E-cigarette Policy Scan in its 2018 report reviewed the situation in 98 countries including the following regulatory domains: minimum age to purchase, sale, advertising, promotion and sponsorship, packaging (child safety packaging, health warning labeling, trademark), product regulation (nicotine volume/concentration, safety/hygiene, ingredients/flavors), reporting/notification, vape-free, and tax (25). In 2018, the COP8/FCTC reported that 104 out of 181 Parties did not regulate ENDS (26). All the stakeholders we interviewed agreed on setting a minimum age for the purchase of e-cigarettes. In a survey conducted by the HPA, 92.6% of respondents agreed that “*sales to minors should be prohibited”* (18). Our interviewees also expressed this high level of support. We must consider, however, that banning cigarette sales to minors is not very effective (27), and this also might apply to banning e-cigarette sales. Moreover, it could have the very unwanted effect of increasing tobacco smoking in minors (28–30).

Stakeholders in Taiwan need to come to some consensus on the level of youth vaping above which action is required. If no vaping at all is tolerable among youth, then drastic anti-vaping measures must be enforced. However, measures to eliminate such competition will protect the combustible cigarette market. Some vaping in youth will have to be tolerated to maximize the number of adult smokers who switch to non-combustible products. Protecting youths against hypothetical gateways should not be given absolute priority over helping adult smokers to quit (31).

Adding labels notifying consumers of content and nicotine level was a measure that was welcome by stakeholders. The TFDA published a standard testing method for nicotine level in e-liquids. Currently, any nicotine detected in e-liquid will be regarded illegal. The European Union treats e-cigarettes and e-liquids containing more than 20 mg/ml nicotine as a medicinal or pharmaceutical product (12); but this upper limit is not grounded in empirical evidence, and precludes European vapers from using high-nicotine liquids (e.g., 50 mg/mL) that deliver a satisfactory quantity of nicotine in a very small volume of vapor, thereby reducing exposure to other vapor constituents. The addition of labels or leaflets informing about nicotine levels and contents is supported by consumers' right to know, and by the need to monitor labeling consistency and packaging compliance. Even though no e-cigarette application has yet been filed, the TFDA should soon publish guidelines on labels and maximum nicotine levels.

Some studies suggest that, in Europe and in the USA, banning e-cigarette use in public places receives public support (2, 3). Almost all our interviewees, including vapers who did not think secondhand smoke is an issue, supported banning e-cigarettes in public places. This result is possibly explained by the fact that, for over 20 years, the THPA has protected by-standers from tobacco smoke in most public places in Taiwan. However, vaping bans may have adverse effects, if vapers must routinely rub shoulders with smokers in smoking/vaping areas, given that it could increase former smokers' risk of relapse and never-smokers' risk of smoking initiation. This potential outcome was not mentioned by any of our interviewed stakeholders.

E-cigarettes have been regulated in Taiwan by existing acts since 2009, and reported violations have increased since 2014 (32). Over this period of time, the adult smoking rate in Taiwan has dropped, from 21.9% in 2008 to 17.1% in 2015; youth smoking rates have decreased as well (33). The Royal College of Physicians, National Academies of Sciences, Engineering, and Medicine and Public Health England reports all agree that e-cigarettes might help smokers quit, even though the quality of the evidence is low (34–36). To our knowledge, there is no documented evidence demonstrating that any significant switch from cigarettes to e-cigarettes has occurred in Taiwan.

The vapers we interviewed all were ex-smokers, and they failed to point out the potential negative effects of applying the same rules to e-cigarettes as combustible tobacco cigarettes.

Shortly after we completed our data collection, in January 2017, the HPA proposed an amended draft of the THPA that included e-cigarettes (17). Though ideally all stakeholders should be involved to generate the most prudent regulations for e-cigarettes (37), the amended THPA was discussed among multi-sectoral ministry personnel and other stakeholders, short of including vapers. The proposed amendments were sent to the Legislative Yuan on 29 December 2017 after receiving approval from the Prime Minister on 21 December 2017. We anticipate that, under this draft, e-cigarettes will no longer be available for recreational use; only those that are approved for medicinal purposes will be manufactured, imported, and sold. As a result, the combustible cigarette market will receive *de facto* governmental protection against competition from less dangerous, non-combustible products.

### Strengths and Limitations of This Study

The small sample and its lack of representativeness are among the limitations of this study. We identified a variety of stakeholders involved in e-cigarette policy, legislation, implementation, business, legal experts, and professionals. However, we could have enrolled a more representative sample by including legislators who pushed for amending the THPA to cover e-cigarette as documented in legislative report (38).

One strength of our study was to include a pro-e-cigarette group to balance the perspectives. There was a geographic imbalance in our sample, as most interviewees were from the northern region of Taiwan, which is more urbanized than the south. Another issue in our sample is that most participants had either a university or post-graduate education, though we did not collect other socio-demographic information, such as age and income. Vapers and persons involved in e-cigarette businesses were over-represented in our sample; they spoke from both business owner and user perspectives, and generally were more willing to talk than other interviewees. We would have more confidence in our findings if we had enrolled a larger, and more representative sample. Another weakness is that six out of 14 interviews were not recorded but were manually transcribed during the interviews.

Nevertheless, our findings provide a reference for proposing amendments to e-cigarette legislation in both national and international community facing same issue. In addition, our findings on specific policy issues are useful, kin terms of identifying the need for information and education among policy makers, health professionals and the public in Taiwan, and for debating potential e-cigarette regulations.

This is one of few qualitative researches in Asia (and the first in Taiwan) exploring e-cigarette stakeholders' opinions to e-cigarette regulation. Even though our findings can be useful for other Asian countries, they may not be generalizable because tobacco control policy and other contextual elements differ across countries.

## Conclusions

The interviews we conducted suggest that, in Taiwan, the current legal system of regulating e-cigarettes has weaknesses. Authorities face pressure from both anti-tobacco and pro-e-cigarette groups. Stakeholders generally agree that a systematic legal framework is needed for e-cigarettes, especially those containing nicotine; and most agree with applying many of the same control measures for e-cigarettes as for conventional tobacco products. However, this view does not reflect the continuum of risks incurred by users of the different products. Our respondents did not seem to recognize that applying the same rules to cigarettes and e-cigarettes could exert adverse effects, if such legislation reduces the number of smokers who switch to non-combustible products; and if it protects the combustible cigarette market against competition from reduced-risk products.

## Data Availability Statement

The datasets for this manuscript are not publicly available because of conditions agreed with the participants to protect the participant stakeholders. Requests to access the datasets should be directed to C-SS, chinsshih@gmail.com.

## Ethics Statement

This study received a Certificate of REC Approval from the Research Ethics Committee of the National Taiwan Normal University.

## Author Contributions

C-SS and J-FE designed the protocol. C-SS conducted and transcribed the interviews, analyzed the data, and wrote the first manuscripts. All authors approved the manuscript.

### Conflict of Interest

The authors declare that the research was conducted in the absence of any commercial or financial relationships that could be construed as a potential conflict of interest.
